# Supplementary material for: Optimising pacemaker therapy and medical therapy in pacemaker patients for heart failure: protocol for the OPT-PACE randomised controlled trial
Source: BMJ Open. 2019 Jul 17;9(7):e028613. doi: 10.1136/bmjopen-2018-028613 (PMC6661620; doi:10.1136/bmjopen-2018-028613)
Supplement: Author's manuscript [file bmjopen-2018-028613.draft_revisions.pdf]

BMJ Open

BMJ Open is committed to open peer review. As part of this commitment we make the peer review history of every article we publish publicly available.

When an article is published we post the peer reviewers' comments and the authors' responses online. We also post the versions of the paper that were used during peer review. These are the versions that the peer review comments apply to.

The versions of the paper that follow are the versions that were submitted during the peer review process. They are not the versions of record or the final published versions. They should not be cited or distributed as the published version of this manuscript.

BMJ Open is an open access journal and the full, final, typeset and author-corrected version of record of the manuscript is available on our site with no access controls, subscription charges or pay-per-view fees (http://bmjopen.bmj.com).

If you have any questions on BMJ Open's open peer review process please email info.bmjopen@bmj.com

BMJ Open

Optimising pacemaker therapy and medical therapy in pacemaker patients for heart failure - the OPT-PACE randomised controlled trial

Journal:	*BMJ Open*
Manuscript ID	bmjopen-2018-028613
Article Type:	Protocol
Date Submitted by the Author:	17-Dec-2018

Complete List of Authors:	Paton, Maria; University of Leeds, Leeds Institute of Cardiovascular and Metabolic Medicine<br>Gierula, John; University of Leeds, Leeds Institute of Cardiovascular and Metabolic Medicine<br>Jamil, Haqeel; University of Leeds, Leeds Institute of Cardiovascular and Metabolic Medicine<br>Lowry, Judith; University of Leeds, Leeds Institute of Cardiovascular and Metabolic Medicine<br>Byrom, Rowena; University of Leeds, Leeds Institute of Cardiovascular and Metabolic Medicine<br>Gillott, Richard; Leeds Teaching Hospitals NHS Trust<br>Chumun, Hemant; University of Leeds, Leeds Institute of Cardiovascular and Metabolic Medicine<br>Cubbon, R.M.; University of Leeds,<br>Cairns, David; University of Leeds, Leeds Institute of of Clinical Trials Research<br>Stocken, Deborah; University of Leeds, Leeds Institute of Clinical Trials Research<br>Kearney, Mark; University of Leeds, Leeds Institute of Cardiovascular and Metabolic Medicine<br>Witte, Klaus; University of Leeds Leeds Institute of Health Sciences, Faculty of Medicine and Health; Leeds General Infirmary, Cardiology

Keywords:	pacemaker, Optimal medical therapy, Heart failure < CARDIOLOGY, randomised controlled trial, Pacing & electrophysiology < CARDIOLOGY

SCHOLARONE™ Manuscripts

Optimising pacemaker therapy and medical therapy in pacemaker patients for heart failure - the OPT-pace randomised controlled trial

Maria F Paton[1]
John Gierula[1]
Haqeel A Jamil[1]
Judith E Lowry[1]
Rowena Byrom[1]
Richard G Gillott[2]
Hemant Chumun[1]
Richard M Cubbon[1]
David A Cairns[3]
Deborah D Stocken[3]
Mark T Kearney[1]
Klaus K Witte[1*]

For peer review only

Running title: Heart failure in pacemaker patients
ClinicalTrials.gov registration number: NCT01819662
Word count for Main body: 2917
Number of tables: 0
Number of figures: 2
Number of supplementary tables: 0
Number of supplementary figures: 0

[1] Leeds Institute of Cardiovascular and Metabolic Medicine, University of Leeds, Leeds, UK
[2] Leeds Teaching Hospitals NHS Trust, Department of Cardiology, Leeds, UK
[3] Leeds Institute of Clinical Trials Research, University of Leeds, Leeds, UK

* Corresponding author:
Dr Klaus K Witte
Division of Cardiovascular and Diabetes Research,
Multidisciplinary Cardiovascular Research Centre (MCRC)
Leeds Institute of Cardiovascular and Metabolic Medicine
LIGHT building, University of Leeds
Clarendon Way, Leeds, UK, LS2 9JT

Phone: (+44) 113 3926108
E-mail: k.k.witte@leeds.ac.uk

Trial Sponsor : University of Leeds
LIGHT building,
Clarendon Way, Leeds, UK, LS2 9JT

Abstract

Introduction: Permanent artificial pacemaker implantation is a safe and effective treatment for bradycardia and is associated with extended longevity and improved quality of life. However, the most common long-term complication of standard pacemaker therapy is pacemaker-associated heart failure. Pacemaker follow-up is potentially an opportunity to screen for heart failure to assess and optimise patient devices and medical therapy.

Methods and Analysis: The study is a multi-centre, phase-3 randomised trial. The 1200 participants will be people who have a permanent pacemaker for bradycardia for at least 12 months, randomly assigned to undergo a transthoracic echocardiogram with their pacemaker check, thereby tailoring their management directed by left ventricular function, or the pacemaker check alone, continuing with routine follow-up. The primary outcome measure is time to all-cause mortality or heart failure hospitalisation. Secondary outcomes include external validation of our risk stratification model to predict onset of heart failure and quality of life assessment.

Ethics and Dissemination: Discussion: The trial design and protocol has received national ethical approval (12/YH/0487) and is registered on ClinicalTrials.gov (NCT01819662).

The results of this randomised trial will provide evidence on the effectiveness of optimising medical therapy based on echocardiographic and pacing parameters and its role in potentially reducing the incidence of heart failure in pacemaker patients.

Keywords: Pacemaker, optimal medical therapy, heart failure, randomised controlled trial

For peer review only - http://bmjopen.bmj.com/site/about/guidelines.xhtml

Article Summary

Strengths and Limitations of this study

- OPT-PACE is a multicentre, randomised, non-blinded parallel trial funded by the National Institute for Health Research
- Stratification of the participant randomisation is not adopted in this trial due to the large sample size.
- Participant and practitioner blinding are not plausible.
- The control arm consists of current standard of care ensuring trial results are reflective and generalizable.

For peer review only

For peer review only - http://bmjopen.bmj.com/site/about/guidelines.xhtml

Introduction

Permanent artificial pacemaker implantation is a safe and effective treatment for bradycardia,[1] and is associated with extended longevity [2] and improved quality of life.[3] An estimated 350,000 people in the UK have a pacemaker, with over 40,000 new implants per year.

However, long term right ventricular (RV) pacing has been linked to adverse left ventricular (LV) remodeling, [4, 5] such that the most common long-term complication of standard pacemaker therapy is pacemaker-associated chronic heart failure (CHF) due to left ventricular systolic dysfunction (LVSD) [6-8]. Whilst up to 2-3% of the general population have CHF, the condition is much more common in pacemaker patients with a prevalence up to 50% [7, 9] and 12% of people admitted with acute decompensated heart failure (HF) (4% with a de novo admission for heart failure) have a pacemaker [10].

It is clear that right ventricular pacing alone can lead to LVSD, however the risk is especially high in people requiring a high proportion of ventricular pacing, those with diabetes mellitus, previous myocardial infarction and raised creatinine [11]. Data examining the relationship between pacemaker use and cardiac dysfunction predominantly originate from retrospective cross-sectional analysis or secondary analyses [12-15]. We provided the first evidence that reducing RV pacing through careful reprogramming is associated with an improvement in LV function without affecting quality of life or functional capacity,[9] confirming somewhat the suggestion that RV pacing is contributory to LV dysfunction in pacemaker patients and not just a bystander in a multi-morbid patient population.

For peer review only - http://bmjopen.bmj.com/site/about/guidelines.xhtml

Although there is increasing recognition of the axis of RV pacing and LVSD, it remains the case that HF is frequently overlooked in the pacemaker population with symptoms often ascribed to pacemaker syndrome, chronotropic incompetence, or co-morbidities [6]. HF and undiagnosed left ventricular dysfunction have a major effect on mortality and morbidity amongst pacemaker patients [16-18]. Since HF management accounts for approximately 2% of the healthcare budget in many developed countries, more than 60% of which relates to hospitalisation costs [19], optimal management of people with HF whether or not they have a pacemaker is a key strategy for controlling healthcare costs while increasing quality of life [20].

There are however, no data on the potential benefits of screening people with pacemakers for heart failure and whilst there are now a series of algorithms to limit RV pacing [21-23], no clear strategy for the use of these is outlined in current guidelines. This situation is compounded by a lack of published evidence around the benefits of medical management of pacemaker related HF since people with pacemakers were excluded from most of the large heart failure studies.

Prospective randomised studies of pacemaker follow-up programs to risk stratify patients and optimise management have not yet been of adequate size and duration to assess the clinical effectiveness of such an approach in a real-world general healthcare setting. Pacemaker follow-up is a rarely realised opportunity to screen for HF and assess and optimise patient devices and medical therapy.[24]

For peer review only - http://bmjopen.bmj.com/site/about/guidelines.xhtml

The OPT-PACE (OPTimising PACEmaker therapy) trial has been designed to address these issues and quantify time to all-cause mortality or heart failure hospitalisation, as well as validate a HF risk stratification model based on simple variables available in pacemaker clinic. Secondary aims are to establish whether NT-proBNP improves this risk stratification model. Subsequently, we aim to provide data on the effects of establishing the presence of left ventricular systolic dysfunction on medical therapy, device programming and patient quality of life.

Pilot Data

Risk model

The design of the OPT-PACE trial is informed by an observational cohort [25] including 491 patients listed for a pacemaker generator replacement in a single tertiary centre (Leeds Teaching Hospitals NHS Trust) invited for pacing therapy information, diagnostic pacing data and an echocardiogram.

Of this cohort, 40% had an LV ejection fraction of <50%, which was much higher (59%) in those with >80% RV pacing (p<0.001), demonstrating that patients with RV pacemakers have a high prevalence of LV systolic dysfunction. After a mean follow-up time of 668 days, 56 patients (12%) had died or been hospitalized for heart failure.

Multivariable analyses identified a number of simple clinical variables; high percentage RV pacing, high serum creatinine, and previous myocardial infarction as potential independent predictors of LV systolic dysfunction to identify patients who may benefit from a more

For peer review only - http://bmjopen.bmj.com/site/about/guidelines.xhtml

comprehensive review [11]. This risk model requires external validation planned within the OPT-PACE trial.

Intervention Development

The design of the pacemaker optimisation intervention used in the OPT-PACE trial is based on data from an observational cohort of 66 patients, referred for pacemaker generator replacement, recruited consecutively from a single tertiary centre (Leeds Teaching Hospitals NHS Trust)[25]. All patients recruited had been referred for pacemaker generator replacement. Exclusion criteria were inability to consent, underlying complete heart block, a life expectancy of <1 year in the opinion of the clinician (e.g. terminal malignancy), the presence of a device-relate complication, or those with known left ventricular ejection fraction <50%.

In patients recruited with avoidable RV pacing, a pre-specified protocol was followed to reduce this as outlined in figure 1 including a reduction in the diurnal base rate (BR) to 50 beats/min with nocturnal rate, or hysteresis to 40beats/min along with the deactivation of rate-adaptive pacing. In addition, where sinus rhythm and heart block had been documented, the atrioventricular (AV) delays were extended or a device with RV avoidance algorithms was implanted. The patients were re-assessed 6 months post randomisation. The primary endpoint was left ventricular ejection fraction (LVEF) at follow-up calculated as an average over 3 non-paced, normally conducted beats using modified Simpson's method as per ASE guidelines [26]. The protocol was tolerated in all but two subjects in whom rate-responsive pacing was reactivated. Nevertheless, application of the protocol reduced absolute RV pacing percentage by a mean of 49% (95%CI 41-57%; p<0.0001) from baseline and resulted in a mean

For peer review only - http://bmjopen.bmj.com/site/about/guidelines.xhtml

absolute improvement in LVEF of 6% (95%CI: 2-8%; p<0.0001). This occurred without reduction in exercise capacity, NT-proBNP, or quality of life. An association was seen between the reduction in RV pacing and magnitude of change in LVEF (p=0.04). The intervention was considered safe to use without adaptation in OPT-PACE.

Methods and Analysis

OPT-PACE is a multicentre, randomised, non-blinded parallel trial funded by the National Institute for Health Research (NIHR) in the UK (NIHR-CS-2012-032) and registered with the Clinical Trials registry (NCT01819662). The trial design and protocol has received approval from necessary regulatory and ethics boards as well as an independent trial steering committee.

The trial will recruit 1200 patients, which began in June 2013 and all patients will be followed up for a minimum of 12 months.

Trial participation may be terminated via voluntary refusal to continue at any time, or through significant clinical deterioration assessed by medical staff. Participants suffering from trial-related problems or adverse events (AE's) may obtain medical treatment as compensation, and the trial is insured to compensate.

Patients

Patients are recruited from one tertiary centre (Leeds Teaching Hospitals Trust) and two district centres (Harrogate District Foundation Trust and Bradford District Trust). Participants

For peer review only

will have an implantable pacemaker for bradycardia for at least 12 months according to current accepted clinical guidelines [27] and be able to provide written informed consent. Participants will be excluded if they have an implantable cardioverter defibrillator or cardiac resynchronisation device, are less than 18 years old, pregnant, awaiting heart transplantation or have a severe co-morbidity with life expectancy of <1 year. We will also exclude people with significant cognitive impairment and any already under the care of HF services.

Randomisation and Interventions

Potential participants will receive a thorough explanation of the process of the trial, be notified of appointments via post, and associated travel costs will be available to maximise compliance. Potential participants will be asked to voluntarily provide written informed consent prior to randomisation in line with the Declaration of Helsinki 2002.

Consecutive pacemaker clinic attendees agreeing to participate will be enrolled and randomised centrally using a study-specific electronic system. Patients are randomised in a 1:1 ratio to 1) standard care pathway or 2) enhanced care pathway (intervention) (figure 2). Usual care pathways follow NICE guidelines [28] and patients will undergo routine, usually annual, pacemaker follow-up. Enhanced care pathway consists of enhanced or optimised care as pre-determined by each recruiting site. The echocardiographically informed pathways will consist either of a primary care driven management plan based upon the echocardiogram which will be forwarded to the primary care team (enhanced care) or a comprehensive care package consisting of optimised programming and medical therapy coordinated by a regional heart failure service (optimised care). This latter package will include the RV pacing avoidance algorithm (figure 1) and in those with LVSD (LVEF<50%) medical therapy following the NICE

For peer review only - http://bmjopen.bmj.com/site/about/guidelines.xhtml

guidelines for the management of chronic heart failure [29]. In the presence of LVSD, patients recruited at the tertiary centre will be referred to HF clinic, in the district centres, an echocardiogram report will be sent to the patients' general practitioner.

Blinding

Practitioners and participants in this trial cannot be blinded to allocation of treatment due to the nature of the intervention. By not participating in baseline assessments or medical and device optimisation and having no prior knowledge of participant allocation, the outcome assessor will remain blinded and thereby safe from detection bias [30]. Unblinding of the assessors will only be permitted in certain circumstances, for example, when the information is demanded to ensure appropriate management of the participants (e.g. Serious AE's).

Outcome Measures

The primary outcome measure is time from randomisation to date of first event of all-cause mortality or heart failure hospitalisation as assessed by the Endpoint Review Committee. The endpoint review committee will ensure the quality of conduct and assess protocol deviations due to the low risk associated with intervention (echocardiography). They will monitor the safety of the trial and have unblinded access to efficacy data should it be required.

The main secondary outcome measure is the validation of a model to risk stratify people with a pacemaker for prevalent or future heart failure using simple clinical and pacing variables to predict the presence of left ventricular systolic dysfunction at baseline and subsequently the combined outcome of HF hospitalisation and mortality at one year. Validation will be

For peer review only - http://bmjopen.bmj.com/site/about/guidelines.xhtml

achieved by assessing against pre-specified criteria, formed from the confidence intervals of the initial pilot data analysis of the model [9].

Other secondary outcome measures are to determine 1) the utility of BNP testing as a way to improve risk score for prevalent left ventricular systolic dysfunction and future admissions, 2) hospitalisation rates and mortality across randomised arms, 3) utility of a diagnosis of cardiac dysfunction on achieving standard of care medications and doses and 4) quality of life as measured by the EuroQol (EQ5D) at baseline and 12 months post enrollment.

All adverse events related to the interventions will be recorded and monitored until resolved. Medical personnel will decide if continued trial participation is feasible based on these reports, with the final decision made by the participant.

Data Collection

A clinical research co-ordinator (CRC) will collect information on demographic characteristics such as: gender, age, vital signs, height and weight and medication history. The patients' medical history will be taken along with pacemaker data (implant date, implanted device, programmed pacemaker mode, base rate, and cumulative pacing percentage), and blood draw will be obtained. Biological specimens will be analysed and stored in the on-site research pathology laboratory. Patients randomised to intervention will undergo a baseline transthoracic echocardiogram which will be assessed by the CRC. All data will be stored anonymously and will be locked once complete.

To ensure protocol adherence, a single research team will undertake all study-specific activity and be regularly monitored.

For peer review only - http://bmjopen.bmj.com/site/about/guidelines.xhtml
For peer review only

Sample size

The trial is designed to detect a reduction in hospitalisation or mortality rate of 7.5% at one year in patients identified with cardiac dysfunction from 15% anticipated in patients randomised to the standard pathway [9]. Given approximately one third of patients in both randomised arms are estimated to have cardiac dysfunction, a 7.5% reduction would be diluted to a 9% overall reduction in the enhanced pathway arm. To detect a reduction in events from 15% to 9% (equivalent to a hazard ratio equal to 0.58) using log-rank analysis with an overall type 1 error rate of 0.05 (two-sided analysis) and a power of 0.90, requires a total of 146 events to be observed in at least 1070 participants (nQuery Advisor assuming 18 month recruitment) inflated to 1200 in anticipation of minimal drop-out.

Statistical Analysis Plan

Primary analysis will be performed using the pre-specified endpoints in the intention-to-treat population (all randomised patients). . Time to event will be summarised across randomised groups using Kaplan-Meier estimates, hazard ratios and comparison across groups using log-rank tests, reporting 12 month survival.

Secondary analyses will be based on estimating adjusted treatment effects through Cox Proportional Hazards regression model the influence of baseline patient characteristics on outcome. These variables include age, gender, site, co-morbidities, type of device, NYHA class, medication, blood measures and underlying rhythm. A post-hoc exploratory cost-effectiveness analysis will be conducted.

For peer review only - http://bmjopen.bmj.com/site/about/guidelines.xhtml
For peer review only

Quality of life data is collected at 2 time points and will be summarised graphically, conditional on patient survival. Quality adjusted time to event analysis may be appropriate to report quality adjusted estimates of 12-month survival.

External validation of the risk model will be based on fitting the published model to the OPT-PACE trial data to report its goodness of fit. Data will be pooled, and stratified by study, to remodel parameter estimates with the aim of improving precision.

Patient and Public Involvement

The research question developed as a large number of pacemaker patients were seen in HF clinic with deterioration in their ventricular function and symptoms who were followed-up annually in pacemaker clinic and yet, these had not been detected. The study was initially discussed with a well-established local patient and public involvement (PPI) advisory group (AG) consisting of cardiovascular patients and their families. The AG particularly felt that undergoing all study assessments in one day was appealing to potential participants but that the intervention was not a large burden and that it was more important to know the effect on patient survival and the number of times patients were hospitalised, not just whether there was an improvement in heart function from optimised care in terms of outcome measures. Once the final protocol was established, it was reviewed by the PPI-AG along with patient information sheets to ensure the plain English was at an appropriate level. Regular updates will be presented regarding recruitment and any adverse events. Study results will be discussed and an appropriate method of dissemination will be designed together between the AG and researchers to deliver these to participants.

For peer review only - http://bmjopen.bmj.com/site/about/guidelines.xhtml

Ethics and Dissemination

The trial design and protocol have received ethical approval from the South Yorkshire research ethics committee (12/YH/0487) and is registered on Clinicaltrials.gov (NCT01819662). Amendments will be documented.

Trial results will be communicated on a local level to healthcare professionals and patient involvement groups. Results will be published in international peer-reviewed journals with authorship as determined by the international committee of medical journal editors' guidelines [31] and highlighted using social media campaigns. The National Institute of Health Research will be made aware prior to publication but no publication restrictions apply.

Discussion

Patients with permanent pacemakers are at increased risk of prevalent heart failure with a reduced quality of life and incident HF leading to hospitalisations and a poorer prognosis. Despite increasing recognition of this, and the development of device-based options to limit unnecessary ventricular pacing, patients with permanent pacemakers rarely undergo any assessment to identify prevalent HF or their future risk of HF. Pacemaker follow-up services have remained resolutely technical with intervals based upon historical risk of pacemaker device failure rather than focusing on patient requirements in the face of negligible pacemaker failure rates and extended battery longevity.

A validated tool combining clinical and technical data by which patients' risk of the one major complication of pacemaker therapy - heart failure - could be assessed, allowing individualised follow-up intervals has the potential to substantially improve patient experience whilst also

For peer review only

For peer review only - http://bmjopen.bmj.com/site/about/guidelines.xhtml

offering the opportunity to focus screening and lead to the initiation of optimal therapy. Although there are several clinical and pacemaker-related features linked to the presence of cardiac dysfunction, no validated risk score exists to select those that should be referred for assessment.

The management pathways used in this trial will offer individualised healthcare, and establish the relative benefits of primary, secondary and tertiary expertise and interventions. Our previous data have shown that reprogramming to avoid RV pacing is safe and well tolerated by patients. Combining this with medical therapy for HF in collaboration with a HF service as a new model of care for people with pacemakers is feasible and could improve their quality of life and overall prognosis whilst also being highly cost-effective. We aim to test the clinical effectiveness of this approach when applied to a large number of patients at multiple sites.

Conclusions

OPT-PACE is an important randomised controlled trial, supported by non-industry funding, that will investigate whether assessing left ventricular function to inform pacemaker programming and medical decisions, can improve the morbidity and mortality in pacemaker patients, compared with usual care.

The results will have implications for the stratification of pacemaker patients and the organisation of diagnostic and therapeutic pathways to reduce the incidence of HF in this population.

Abbreviations

For peer review only - http://bmjopen.bmj.com/site/about/guidelines.xhtml

BR: Base rate; CHF: Chronic heart failure; EQ-5D: EuroQol 5-Dimension; HF: Heart failure; LV: Left ventricle; LVEF: Left ventricular ejection fraction; LVSD: Left ventricular systolic dysfunction; NT-proBNP: N-terminal prohormone of brain natriuretic peptide; RV: Right ventricular

Authors Contributions

KW and MP researched the topic and devised the study. MP and KW provided the first draft of the manuscript. DDS and DAC provide statistical oversight. All other co-authors contributed equally to data collection and manuscript preparation.

Acknowledgements

The authors would like to express their appreciation for the valuable contributions of participants with a pacemaker who are, or will, participate in this trial, the PPI-AG for their practical and insightful suggestions, and for the consistent administrative support of Mrs Andrea Marchant and Miss Lisa Trueman.

Funding Statement

The research is supported by the National Institute for Health Research (NIHR) infrastructure at Leeds, and a NIHR clinician scientist fellowship (NIHR-CS-2012-032). The views expressed are those of the author(s) and not necessarily those of the NHS, the NIHR or the Department of Health. This research was supported by the National Institute for Health Research Leeds Clinical Research Facility.

Endpoint Review Committee

For peer review only

Members: KW, MTK, MP, JF, 2 heart failure specialist nurses

Competing Interests Statement

MP holds a NIHR clinical doctoral fellowship outside of this work. JG holds a NIHR postdoctoral fellowship outside of this work. RMC holds a fellowship with the BHF. MTK holds a BHF chair. KKW holds an NIHR clinician scientist award.

For peer review only

Figure Legend

Figure 1: Ventricular pacing avoidance protocol [9]

Figure 2: OPT-PACE study protocol

References

1. Epstein, A.E., et al., *2012 ACCF/AHA/HRS focused update incorporated into the ACCF/AHA/HRS 2008 guidelines for device-based therapy of cardiac rhythm abnormalities.* Circulation, 2013. **127**(3): p. e283-e352.
2. Shaw, D.B., et al., *Survival in second degree atrioventricular block.* Heart, 1985. **53**(6): p. 587-593.
3. Lamas, G.A., et al., *Quality of life and clinical outcomes in elderly patients treated with ventricular pacing as compared with dual-chamber pacing.* New England Journal of Medicine, 1998. **338**(16): p. 1097-1104.
4. Lee, M.A., et al., *Effects of long-term right ventricular apical pacing on left ventricular perfusion, innervation, function and histology.* Journal of the American College of Cardiology, 1994. **24**(1): p. 225-232.
5. Begg, G., et al., *154 Patients receiving standard pacemaker generator replacements frequently have impaired left ventricular function and exercise intolerance, related to the percentage of right ventricular pacing.* Heart, 2011. **97**(Suppl 1): p. A86-A86.
6. Sweeney, M.O., et al., *Adverse effect of ventricular pacing on heart failure and atrial fibrillation among patients with normal baseline QRS duration in a clinical trial of pacemaker therapy for sinus node dysfunction.* Circulation, 2003. **107**(23): p. 2932-2937.
7. Thackray, S.D., et al., *The prevalence of heart failure and asymptomatic left ventricular systolic dysfunction in a typical regional pacemaker population.* European Heart Journal, 2003. **24**(12): p. 1143-1152.
8. ZHANG, X.H., et al., *New-onset heart failure after permanent right ventricular apical pacing in patients with acquired high-grade atrioventricular block and normal left ventricular function.* Journal of cardiovascular electrophysiology, 2008. **19**(2): p. 136-141.
9. Gierula, J., et al., *Pacing-associated left ventricular dysfunction? Think reprogramming first!* Heart, 2014. **100**(10): p. 765-769.
10. Nieminen, M.S., et al., *EuroHeart Failure Survey II (EHFS II): a survey on hospitalized acute heart failure patients: description of population.* European heart journal, 2006. **27**(22): p. 2725-2736.
11. Gierula, J., et al., *Patients with long-term permanent pacemakers have a high prevalence of left ventricular dysfunction.* Journal of Cardiovascular Medicine, 2015. **16**(11): p. 743-750.
12. Shen, W.-K., et al., *Long-term survival after pacemaker implantation for heart block in patients≥ 65 years.* The American journal of cardiology, 1994. **74**(6): p. 560-564.
13. JELIĆ, V., et al., *Survival in 1,431 pacemaker patients: prognostic factors and comparison with the general population.* Pacing and Clinical Electrophysiology, 1992. **15**(2): p. 141-147.
14. Mayosi, B.M., F. Little, and R.N.S. MILLAR, *Long-term survival after permanent pacemaker implantation in young adults: 30 year experience.* Pacing and clinical electrophysiology, 1999. **22**(3): p. 407-412.
15. Sweeney, M.O., et al., *Reduced ejection fraction, sudden cardiac death, and heart failure death in the mode selection trial (MOST): implications for device selection in elderly patients with sinus node disease.* Journal of cardiovascular electrophysiology, 2008. **19**(11): p. 1160-1166.

For peer review only - http://bmjopen.bmj.com/site/about/guidelines.xhtml

16. Shen, W.-K., et al., *Survival and functional independence after implantation of a permanent pacemaker in octogenarians and nonagenarians: a population-based study.* Annals of internal medicine, 1996. **125**(6): p. 476-480.
17. Brunner, M., et al., *Long-term survival after pacemaker implantation: Prognostic importance of gender and baseline patient characteristics.* European Heart Journal, 2004. **25**(1): p. 88-95.
18. Jahangir, A., et al., *Relation between mode of pacing and long-term survival in the very elderly.* Journal of the American College of Cardiology, 1999. **33**(5): p. 1208-1216.
19. Braunschweig, F., M.R. Cowie, and A. Auricchio, *What are the costs of heart failure?* Europace, 2011. **13**(suppl_2): p. ii13-ii17.
20. Purdy, S., et al., *Ambulatory care sensitive conditions: terminology and disease coding need to be more specific to aid policy makers and clinicians.* Public health, 2009. **123**(2): p. 169-173.
21. Kolb, C., et al., *Reduction of right ventricular pacing with advanced atrioventricular search hysteresis: results of the PREVENT study.* Pacing and Clinical Electrophysiology, 2011. **34**(8): p. 975-983.
22. Sweeney, M., *Search AV Extension and Managed Ventricular Pacing for Promoting Atrioventricular Conduction (SAVE PACe) Trial. Minimizing ventricular pacing to reduce atrial fibrillation in sinus-node disease.* N Engl J Med, 2007. **357**: p. 1000-1008.
23. DAVY, J., et al., *Near elimination of ventricular pacing in SafeR mode compared to DDD modes: a randomized study of 422 patients.* Pacing and clinical electrophysiology, 2012. **35**(4): p. 392-402.
24. Marinskis, G., et al., *Practices of cardiac implantable electronic device follow-up: results of the European Heart Rhythm Association survey.* Europace, 2012. **14**(3): p. 423-425.
25. Anonymous, *Technology eases living with heart failure. New implantable devices, stem cell therapy, and monitoring techniques may improve length and quality of life.* Health News, 2005. **11**(2): p. 7-8.
26. Lang, R.M., et al., *Recommendations for cardiac chamber quantification by echocardiography in adults: an update from the American Society of Echocardiography and the European Association of Cardiovascular Imaging.* Journal of the American Society of Echocardiography, 2015. **28**(1): p. 1-39. e14.
27. Excellence, N.I.f.H.a.C., *Dual-chamber pacemakers for symptomatic bradycardia due to sick sinus syndrome without atrioventricular block (TA324)*. 2014, NICE: London.
28. NICE, *Dual-chamber pacemakers for symptomatic bradycardia due to sick sinus syndrome and/or atrioventricular block*. 2017.
29. Excellence, N.I.f.H.a.C., *Chronic heart failure in adults: management*. 2010, NICE: London.
30. Higgins, J.P.T., S. Green, and Cochrane Collaboration., *Cochrane handbook for systematic reviews of interventions*. 2011, Cochrane Collaboration: [Oxford]. p. 1 online resource.
31. Editors, I.C.o.M.J. *Defining the role of authors and contributors*. 2018 [cited 2018 01/02/2018]; Available from: http://www.icmje.org/recommendations/browse/roles-and-responsibilities/defining-the-role-of-authors-and-contributors.html.

For peer review only - http://bmjopen.bmj.com/site/about/guidelines.xhtml

For peer review only

For peer review only - http://bmjopen.bmj.com/site/about/guidelines.xhtml

Figure 1. Right ventricular avoidance algorithm

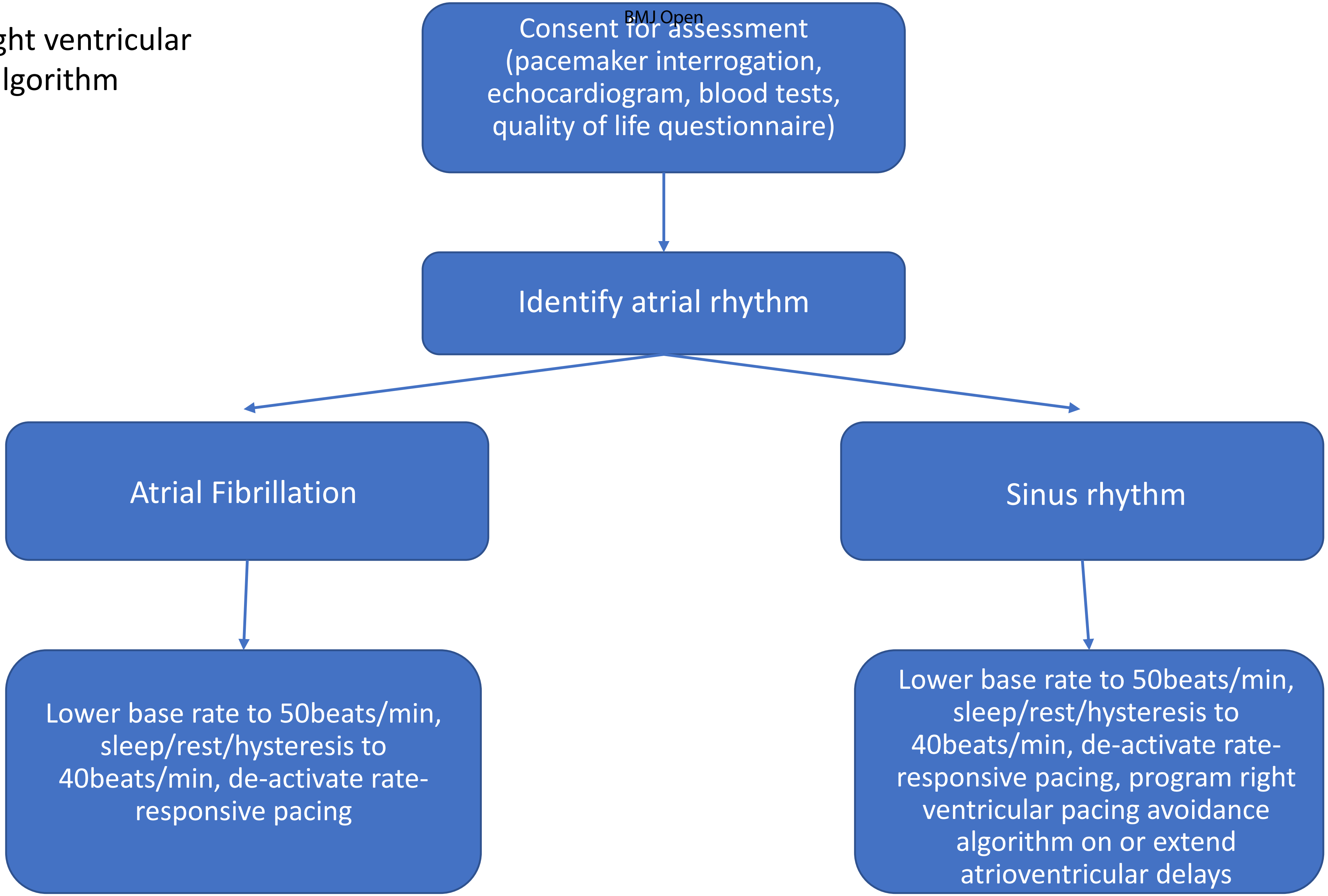

Figure 2. OPT-PACE study protocol

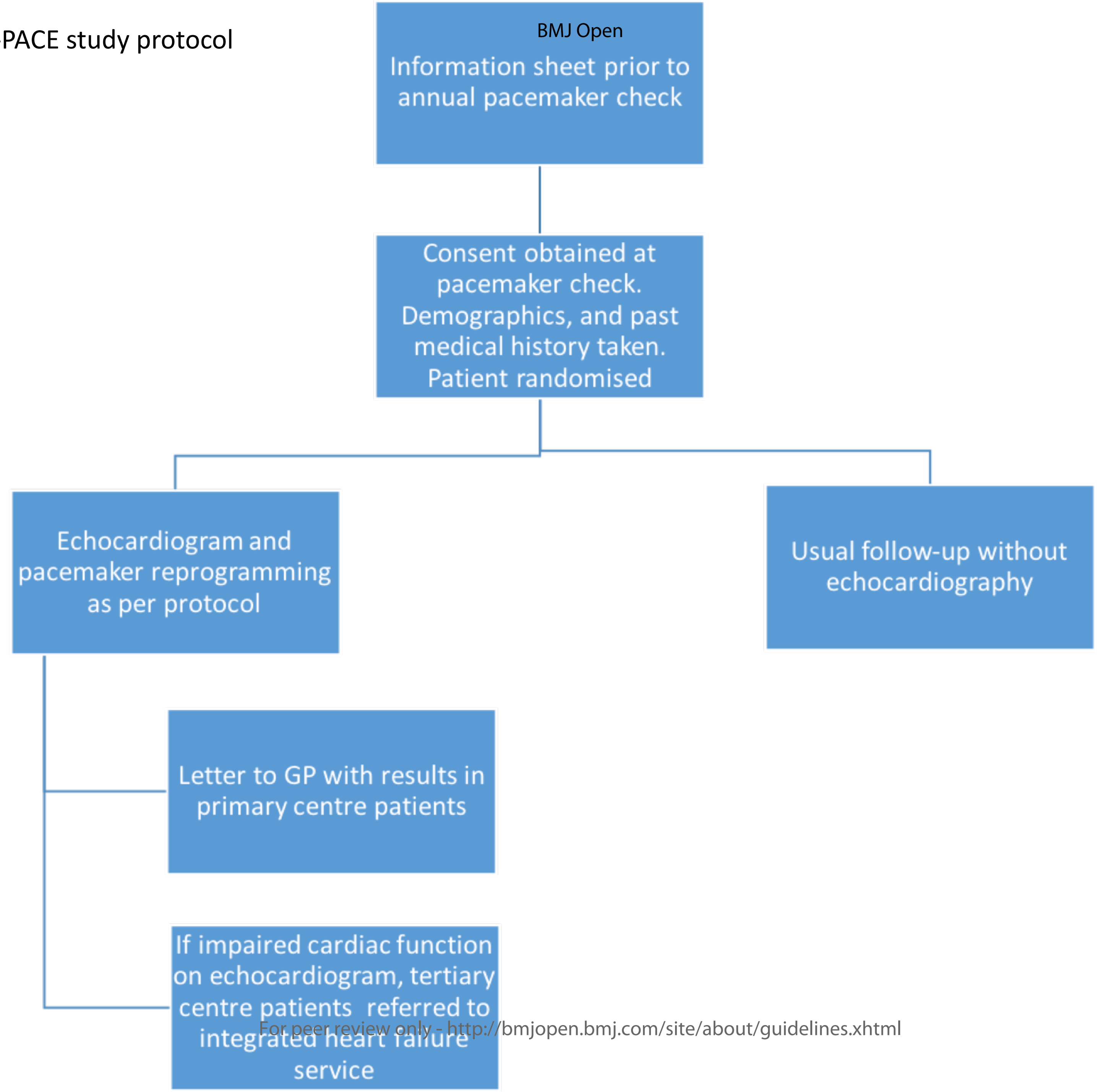

SPIRIT

STANDARD PROTOCOL ITEMS: RECOMMENDATIONS FOR INTERVENTIONAL TRIALS

SPIRIT 2013 Checklist: Recommended items to address in a clinical trial protocol and related documents*

Section/item	Item No	Description	Addressed on page number
**Administrative information**			
Title	1	Descriptive title identifying the study design, population, interventions, and, if applicable, trial acronym	1
Trial registration	2a	Trial identifier and registry name. If not yet registered, name of intended registry	1
	2b	All items from the World Health Organization Trial Registration Data Set	1,
Protocol version	3	Date and version identifier	8
Funding	4	Sources and types of financial, material, and other support	1, 8, 15
Roles and responsibilities	5a	Names, affiliations, and roles of protocol contributors	1, 15
	5b	Name and contact information for the trial sponsor	1
	5c	Role of study sponsor and funders, if any, in study design; collection, management, analysis, and interpretation of data; writing of the report; and the decision to submit the report for publication, including whether they will have ultimate authority over any of these activities	N/A
	5d	Composition, roles, and responsibilities of the coordinating centre, steering committee, endpoint adjudication committee, data management team, and other individuals or groups overseeing the trial, if applicable (see Item 21a for data monitoring committee)	11, 15

For peer review only

**Introduction**			
Background and rationale	6a	Description of research question and justification for undertaking the trial, including summary of relevant studies (published and unpublished) examining benefits and harms for each intervention	4-8
	6b	Explanation for choice of comparators	4-7, 10
Objectives	7	Specific objectives or hypotheses	7
Trial design	8	Description of trial design including type of trial (eg, parallel group, crossover, factorial, single group), allocation ratio, and framework (eg, superiority, equivalence, noninferiority, exploratory)	4-9
**Methods: Participants, interventions, and outcomes**			
Study setting	9	Description of study settings (eg, community clinic, academic hospital) and list of countries where data will be collected. Reference to where list of study sites can be obtained	8,9
Eligibility criteria	10	Inclusion and exclusion criteria for participants. If applicable, eligibility criteria for study centres and individuals who will perform the interventions (eg, surgeons, psychotherapists)	8,9
Interventions	11a	Interventions for each group with sufficient detail to allow replication, including how and when they will be administered	9, 11
	11b	Criteria for discontinuing or modifying allocated interventions for a given trial participant (eg, drug dose change in response to harms, participant request, or improving/worsening disease)	8, 9, 11
	11c	Strategies to improve adherence to intervention protocols, and any procedures for monitoring adherence (eg, drug tablet return, laboratory tests)	11
	11d	Relevant concomitant care and interventions that are permitted or prohibited during the trial	NA
Outcomes	12	Primary, secondary, and other outcomes, including the specific measurement variable (eg, systolic blood pressure), analysis metric (eg, change from baseline, final value, time to event), method of aggregation (eg, median, proportion), and time point for each outcome. Explanation of the clinical relevance of chosen efficacy and harm outcomes is strongly recommended	10,11
Participant timeline	13	Time schedule of enrolment, interventions (including any run-ins and washouts), assessments, and visits for participants. A schematic diagram is highly recommended (see Figure)	8,9, Figure 2

For peer review only - http://bmjopen.bmj.com/site/about/guidelines.xhtml

Sample size	14	Estimated number of participants needed to achieve study objectives and how it was determined, including clinical and statistical assumptions supporting any sample size calculations	11
Recruitment	15	Strategies for achieving adequate participant enrolment to reach target sample size	11-12
**Methods: Assignment of interventions (for controlled trials)**			
Allocation:			
Sequence generation	16a	Method of generating the allocation sequence (eg, computer-generated random numbers), and list of any factors for stratification. To reduce predictability of a random sequence, details of any planned restricton (eg, blocking) should be provided in a separate document that is unavailable to those who enrol participants or assign interventions	8,9
Allocation concealment mechanism	16b	Mechanism of implementing the allocation sequence (eg, central telephone; sequentially numbered, opaque, sealed envelopes), describing any steps to conceal the sequence until interventions are assigned	8,9
Implementation	16c	Who will generate the allocation sequence, who will enrol participants, and who will assign participants to interventions	5-8
Blinding (masking)	17a	Who will be blinded after assignment to interventions (eg, trial participants, care providers, outcome assessors, data analysts), and how	9
	17b	If blinded, circumstances under which unblinding is permissible, and procedure for revealing a participant's allocated intervention during the trial	9,13
**Methods: Data collection, management, and analysis**			
Data collection methods	18a	Plans for assessment and collection of outcome, baseline, and other trial data, including any related processes to promote data quality (eg, duplicate measurements, training of assessors) and a description of study instruments (eg, questionnaires, laboratory tests) along with their reliability and validity, if known. Reference to where data collection forms can be found, if not in the protocol	9
	18b	Plans to promote participant retention and complete follow-up, including list of any outcome data to be collected for participants who discontinue or deviate from intervention protocols	11

For peer review only - http://bmjopen.bmj.com/site/about/guidelines.xhtml

Data management	19	Plans for data entry, coding, security, and storage, including any related processes to promote data quality (eg, double data entry; range checks for data values). Reference to where details of data management procedures can be found, if not in the protocol	8-11
Statistical methods	20a	Statistical methods for analysing primary and secondary outcomes. Reference to where other details of the statistical analysis plan can be found, if not in the protocol	12
	20b	Methods for any additional analyses (eg, subgroup and adjusted analyses)	12
	20c	Definition of analysis population relating to protocol non-adherence (eg, as randomised analysis), and any statistical methods to handle missing data (eg, multiple imputation)	12
**Methods: Monitoring**			
Data monitoring	21a	Composition of data monitoring committee (DMC); summary of its role and reporting structure; statement of whether it is independent from the sponsor and competing interests; and reference to where further details about its charter can be found, if not in the protocol. Alternatively, an explanation of why a DMC is not needed	13
	21b	Description of any interim analyses and stopping guidelines, including who will have access to these interim results and make the final decision to terminate the trial	12
Harms	22	Plans for collecting, assessing, reporting, and managing solicited and spontaneously reported adverse events and other unintended effects of trial interventions or trial conduct	11
Auditing	23	Frequency and procedures for auditing trial conduct, if any, and whether the process will be independent from investigators and the sponsor	11
**Ethics and dissemination**			
Research ethics approval	24	Plans for seeking research ethics committee/institutional review board (REC/IRB) approval	11, 12
Protocol amendments	25	Plans for communicating important protocol modifications (eg, changes to eligibility criteria, outcomes, analyses) to relevant parties (eg, investigators, REC/IRBs, trial participants, trial registries, journals, regulators)	13

Consent or assent	26a	Who will obtain informed consent or assent from potential trial participants or authorised surrogates, and how (see Item 32)	9,11
	26b	Additional consent provisions for collection and use of participant data and biological specimens in ancillary studies, if applicable	N/A
Confidentiality	27	How personal information about potential and enrolled participants will be collected, shared, and maintained in order to protect confidentiality before, during, and after the trial	11
Declaration of interests	28	Financial and other competing interests for principal investigators for the overall trial and each study site	15,16
Access to data	29	Statement of who will have access to the final trial dataset, and disclosure of contractual agreements that limit such access for investigators	11
Ancillary and post-trial care	30	Provisions, if any, for ancillary and post-trial care, and for compensation to those who suffer harm from trial participation	8
Dissemination policy	31a	Plans for investigators and sponsor to communicate trial results to participants, healthcare professionals, the public, and other relevant groups (eg, via publication, reporting in results databases, or other data sharing arrangements), including any publication restrictions	12,13
	31b	Authorship eligibility guidelines and any intended use of professional writers	15
	31c	Plans, if any, for granting public access to the full protocol, participant-level dataset, and statistical code	No plans
**Appendices**			
Informed consent materials	32	Model consent form and other related documentation given to participants and authorised surrogates	N/A can be provided on request
Biological specimens	33	Plans for collection, laboratory evaluation, and storage of biological specimens for genetic or molecular analysis in the current trial and for future use in ancillary studies, if applicable	11,12,13

*It is strongly recommended that this checklist be read in conjunction with the SPIRIT 2013 Explanation & Elaboration for important clarification on the items. Amendments to the protocol should be tracked and dated. The SPIRIT checklist is copyrighted by the SPIRIT Group under the Creative Commons "Attribution-NonCommercial-NoDerivs 3.0 Unported" license.

For peer review only - http://bmjopen.bmj.com/site/about/guidelines.xhtml

For peer review only

6

Optimising pacemaker therapy and medical therapy in pacemaker patients for heart failure – protocol for the OPT-pace randomised controlled trial

Journal:	*BMJ Open*
Manuscript ID	bmjopen-2018-028613.R1
Article Type:	Protocol
Date Submitted by the Author:	30-Apr-2019
Complete List of Authors:	Paton, Maria; University of Leeds, Leeds Institute of Cardiovascular and Metabolic Medicine<br>Gierula, John; University of Leeds, Leeds Institute of Cardiovascular and Metabolic Medicine<br>Jamil, Haqeel; University of Leeds, Leeds Institute of Cardiovascular and Metabolic Medicine<br>Lowry, Judith; University of Leeds, Leeds Institute of Cardiovascular and Metabolic Medicine<br>Byrom, Rowena; University of Leeds, Leeds Institute of Cardiovascular and Metabolic Medicine<br>Gillott, Richard; Leeds Teaching Hospitals NHS Trust<br>Chumun, Hemant; University of Leeds, Leeds Institute of Cardiovascular and Metabolic Medicine<br>Cubbon, R.M.; University of Leeds,<br>Cairns, David; University of Leeds, Leeds Institute of of Clinical Trials Research<br>Stocken, Deborah; University of Leeds, Leeds Institute of Clinical Trials Research<br>Kearney, Mark; University of Leeds, Leeds Institute of Cardiovascular and Metabolic Medicine<br>Witte, Klaus; University of Leeds Leeds Institute of Health Sciences, Faculty of Medicine and Health; Leeds General Infirmary, Cardiology
**Primary Subject Heading**:	Cardiovascular medicine
Secondary Subject Heading:	Cardiovascular medicine
Keywords:	pacemaker, Optimal medical therapy, Heart failure < CARDIOLOGY, randomised controlled trial, Pacing & electrophysiology < CARDIOLOGY

Optimising pacemaker therapy and medical therapy in pacemaker patients for heart failure – protocol for the OPT-pace randomised controlled trial

Maria F Paton[1]
John Gierula[1]
Haqeel A Jamil[1]
Judith E Lowry[1]
Rowena Byrom[1]
Richard G Gillott[2]
Hemant Chumun[1]
Richard M Cubbon[1]
David A Cairns[3]
Deborah D Stocken[3]
Mark T Kearney[1]
Klaus K Witte[1*]

Running title: Heart failure in pacemaker patients
ClinicalTrials.gov registration number: NCT01819662
Word count for Main body: 2917
Number of tables: 0
Number of figures: 2
Number of supplementary tables: 0
Number of supplementary figures: 0

[1] Leeds Institute of Cardiovascular and Metabolic Medicine, University of Leeds, Leeds, UK
[2] Leeds Teaching Hospitals NHS Trust, Department of Cardiology, Leeds, UK
[3] Leeds Institute of Clinical Trials Research, University of Leeds, Leeds, UK

* Corresponding author:
Dr Klaus K Witte
Division of Cardiovascular and Diabetes Research,
Multidisciplinary Cardiovascular Research Centre (MCRC)
Leeds Institute of Cardiovascular and Metabolic Medicine
LIGHT building, University of Leeds
Clarendon Way, Leeds, UK, LS2 9JT

Phone: (+44) 113 3926108
E-mail: k.k.witte@leeds.ac.uk

Trial Sponsor : University of Leeds
LIGHT building,
Clarendon Way, Leeds, UK, LS2 9JT

For peer review only

Abstract

Introduction: Permanent artificial pacemaker implantation is a safe and effective treatment for bradycardia and is associated with extended longevity and improved quality of life. However, the most common long-term complication of standard pacemaker therapy is pacemaker-associated heart failure. Pacemaker follow-up is potentially an opportunity to screen for heart failure to assess and optimise patient devices and medical therapy.

Methods and Analysis: The study is a multi-centre, phase-3 randomised trial. The 1200 participants will be people who have a permanent pacemaker for bradycardia for at least 12 months, randomly assigned to undergo a transthoracic echocardiogram with their pacemaker check, thereby tailoring their management directed by left ventricular function, or the pacemaker check alone, continuing with routine follow-up. The primary outcome measure is time to all-cause mortality or heart failure hospitalisation. Secondary outcomes include external validation of our risk stratification model to predict onset of heart failure and quality of life assessment.

Ethics and Dissemination: The trial design and protocol has received national ethical approval (12/YH/0487) and is registered on ClinicalTrials.gov (NCT01819662).

The results of this randomised trial will be published in international peer-reviewed journals, communicated to healthcare professionals and patient involvement groups, and highlighted using social media campaigns.

Keywords: Pacemaker, optimal medical therapy, heart failure, randomised controlled trial

For peer review only

Article Summary

Strengths and Limitations of this study

- OPT-PACE is a trial independently funded by the National Institute for Health Research
- Stratification of the participant randomisation is not adopted in this trial due to the large sample size.
- Participant and practitioner blinding are not plausible.
- The control arm consists of current standard of care ensuring trial results are reflective and generalizable.

For peer review only

For peer review only - http://bmjopen.bmj.com/site/about/guidelines.xhtml

Introduction

Permanent artificial pacemaker implantation is a safe and effective treatment for bradycardia,[1] and is associated with extended longevity [2] and improved quality of life.[3] An estimated 350,000 people in the UK have a pacemaker, with over 40,000 new implants per year.

However, long term right ventricular (RV) pacing has been linked to adverse left ventricular (LV) remodeling, [4, 5] such that the most common long-term complication of standard pacemaker therapy is pacemaker-associated chronic heart failure (CHF) due to left ventricular systolic dysfunction (LVSD) [6-8]. Whilst up to 2-3% of the general population have CHF, the condition is much more common in pacemaker patients with a prevalence up to 50% [7, 9] and 12% of people admitted with acute decompensated heart failure (HF) (4% with a de novo admission for heart failure) have a pacemaker [10].

It is clear that right ventricular pacing alone can lead to LVSD, however the risk is especially high in people requiring a high proportion of ventricular pacing, those with diabetes mellitus, previous myocardial infarction and raised creatinine [11]. Data examining the relationship between pacemaker use and cardiac dysfunction predominantly originate from retrospective cross-sectional analysis or secondary analyses [12-15]. We provided the first evidence that reducing RV pacing through careful reprogramming is associated with an improvement in LV function without affecting quality of life or functional capacity,[9] confirming somewhat the suggestion that RV pacing is contributory to LV dysfunction in pacemaker patients and not just a bystander in a multi-morbid patient population.

For peer review only

Although there is increasing recognition of the axis of RV pacing and LVSD, it remains the case that HF is frequently overlooked in the pacemaker population with symptoms often ascribed to pacemaker syndrome, chronotropic incompetence, or co-morbidities [6]. HF and undiagnosed left ventricular dysfunction have a major effect on mortality and morbidity amongst pacemaker patients [16-18]. Since HF management accounts for approximately 2% of the healthcare budget in many developed countries, more than 60% of which relates to hospitalisation costs [19], optimal management of people with HF whether or not they have a pacemaker is a key strategy for controlling healthcare costs while increasing quality of life [20].

There are however, no data on the potential benefits of screening people with pacemakers for heart failure and whilst there are now a series of algorithms to limit RV pacing [21-23], no clear strategy for the use of these is outlined in current guidelines. This situation is compounded by a lack of published evidence around the benefits of medical management of pacemaker related HF since people with pacemakers were excluded from most of the large heart failure studies.

Prospective randomised studies of pacemaker follow-up programs to risk stratify patients and optimise management have not yet been of adequate size and duration to assess the clinical effectiveness of such an approach in a real-world general healthcare setting. Pacemaker follow-up is a rarely realised opportunity to screen for HF and assess and optimise patient devices and medical therapy.[24]

For peer review only

For peer review only - http://bmjopen.bmj.com/site/about/guidelines.xhtml

The OPT-PACE (OPTimising PACEmaker therapy) trial has been designed to address these issues and quantify time to all-cause mortality or heart failure hospitalisation, as well as validate a HF risk stratification model based on simple variables available in pacemaker clinic. Secondary aims are to establish whether NT-proBNP improves this risk stratification model. Subsequently, we aim to provide data on the effects of establishing the presence of left ventricular systolic dysfunction on medical therapy, device programming and patient quality of life.

Pilot Data

Risk model

The design of the OPT-PACE trial is informed by an observational cohort [11] including 491 patients listed for a pacemaker generator replacement in a single tertiary centre (Leeds Teaching Hospitals NHS Trust) invited for pacing therapy information, diagnostic pacing data and an echocardiogram.

Of this cohort, 40% had an LV ejection fraction of <50%, which was much higher (59%) in those with >80% RV pacing (p<0.001), demonstrating that patients with RV pacemakers have a high prevalence of LV systolic dysfunction. After a mean follow-up time of 668 days, 56 patients (12%) had died or been hospitalized for heart failure.

Multivariable analyses identified a number of simple clinical variables; high percentage RV pacing, high serum creatinine, and previous myocardial infarction as potential independent predictors of LV systolic dysfunction to identify patients who may benefit from a more

For peer review only

For peer review only - http://bmjopen.bmj.com/site/about/guidelines.xhtml

comprehensive review [11]. This risk model requires external validation planned within the OPT-PACE trial.

Intervention Development

The design of the pacemaker optimisation intervention used in the OPT-PACE trial is based on data from an observational cohort of 66 patients, referred for pacemaker generator replacement, recruited consecutively from a single tertiary centre (Leeds Teaching Hospitals NHS Trust)[9]. All patients recruited had been referred for pacemaker generator replacement. Exclusion criteria were inability to consent, underlying complete heart block, a life expectancy of <1 year in the opinion of the clinician (e.g. terminal malignancy), the presence of a device-relate complication, or those with known left ventricular ejection fraction <50%.

In patients recruited with avoidable RV pacing, a pre-specified protocol was followed to reduce this as outlined in figure 1 including a reduction in the diurnal base rate (BR) to 50 beats/min with nocturnal rate, or hysteresis to 40beats/min along with the deactivation of rate-adaptive pacing. In addition, where sinus rhythm and heart block had been documented, the atrioventricular (AV) delays were extended or a device with RV avoidance algorithms was implanted. The patients were re-assessed 6 months post randomisation. The primary endpoint was left ventricular ejection fraction (LVEF) at follow-up calculated as an average over 3 non-paced, normally conducted beats using modified Simpson's method as per ASE guidelines [25]. The protocol was tolerated in all but two subjects in whom rate-responsive pacing was reactivated. Nevertheless, application of the protocol reduced absolute RV pacing percentage by a mean of 49% (95%CI 41-57%; p<0.0001) from baseline and resulted in a mean absolute improvement in LVEF of 6% (95%CI: 2-8%; p<0.0001). This occurred without

For peer review only - http://bmjopen.bmj.com/site/about/guidelines.xhtml

reduction in exercise capacity, NT-proBNP, or quality of life. An association was seen between the reduction in RV pacing and magnitude of change in LVEF (p=0.04). The intervention was considered safe to use without adaptation in OPT-PACE.

Methods and Analysis

OPT-PACE is a multicentre, randomised, non-blinded parallel trial funded by the National Institute for Health Research (NIHR) in the UK (NIHR-CS-2012-032) and registered with the Clinical Trials registry (NCT01819662). The trial design and protocol has received approval from necessary regulatory and ethics boards as well as an independent trial steering committee.

The trial will recruit 1200 patients, which began in June 2013 and all patients will be followed up for a minimum of 12 months.

Trial participation may be terminated via voluntary refusal to continue at any time, or through significant clinical deterioration assessed by medical staff. Participants suffering from trial-related problems or adverse events (AE's) may obtain medical treatment as compensation, and the trial is insured to compensate.

Patients

Patients are recruited from one tertiary centre (Leeds Teaching Hospitals Trust) and two district centres (Harrogate District Foundation Trust and Bradford District Trust). Participants will have an implantable pacemaker for bradycardia for at least 12 months according to any

For peer review only

For peer review only - http://bmjopen.bmj.com/site/about/guidelines.xhtml

indication in current accepted clinical guidelines [26] and be able to provide written informed consent. Both atrial and ventricular pacing burdens will be documented. Participants will be excluded if they have an implantable cardioverter defibrillator or cardiac resynchronisation device, are less than 18 years old, pregnant, awaiting heart transplantation or have a severe co-morbidity with life expectancy of <1 year. We will also exclude people with significant cognitive impairment and any already under the care of HF services.

Randomisation and Interventions

Potential participants will receive a thorough explanation of the process of the trial, be notified of appointments via post, and associated travel costs will be available to maximise compliance. Potential participants will be asked to voluntarily provide written informed consent prior to randomisation in line with the Declaration of Helsinki 2002.

Consecutive pacemaker clinic attendees agreeing to participate will be enrolled and randomised centrally using a study-specific electronic system. Patients are randomised in a 1:1 ratio to 1) standard care pathway or 2) interventional pathway (figure 2). Usual care pathways follow NICE guidelines [27] and patients will undergo routine, usually annual, pacemaker follow-up. Interventional pathways consists of enhanced or optimised care pre-determined by each recruiting site. The echocardiographically informed pathways will consist either of a primary care driven management plan based upon the echocardiogram which will be forwarded to the primary care team (enhanced care, n=300) if recruited in one of the district centres, or a comprehensive care package consisting of optimised programming and medical therapy co-ordinated by a regional heart failure service (optimised care, n=300) if recruited in the tertiary centre. This interventional package will include the RV pacing

For peer review only

avoidance algorithm (figure 1) and in those with LVSD (LVEF<50%) medical therapy following the NICE guidelines for the management of chronic heart failure [28]. In the presence of LVSD, patients recruited at the tertiary centre will be referred to an outpatient HF clinic (optimized care), and in the district centres an echocardiogram report will be sent to the patients' general practitioner (enhanced care).

Blinding

Practitioners and participants in this trial cannot be blinded to allocation of treatment due to the nature of the intervention. By not participating in baseline assessments or medical and device optimisation and having no prior knowledge of participant allocation, the outcome assessor will remain blinded and thereby safe from detection bias [29]. Unblinding of the assessors will only be permitted in certain circumstances, for example, when the information is demanded to ensure appropriate management of the participants (e.g. Serious AE's).

Outcome Measures

The primary outcome measure is a combined endpoint of time from randomisation to date of first event of all-cause mortality or heart failure hospitalisation as assessed by the Endpoint Review Committee. The endpoint review committee will ensure the quality of conduct and assess protocol deviations due to the low risk associated with intervention (echocardiography). They will monitor the safety of the trial and have unblinded access to efficacy data should it be required.

The main secondary outcome measure is the validation of a model to risk stratify people with a pacemaker for prevalent or future heart failure using simple clinical and pacing variables to

For peer review only - http://bmjopen.bmj.com/site/about/guidelines.xhtml

predict the presence of left ventricular systolic dysfunction at baseline and subsequently the combined outcome of HF hospitalisation and mortality at one year. Validation will be achieved by assessing against pre-specified criteria, formed from the confidence intervals of the initial pilot data analysis of the model [9].

Other secondary outcome measures are to determine 1) the utility of BNP testing as a way to improve risk score for prevalent left ventricular systolic dysfunction and future admissions, 2) hospitalisation rates and mortality across randomised arms, 3) utility of a diagnosis of cardiac dysfunction on achieving standard of care medications and doses and 4) quality of life as measured by the EuroQol (EQ5D) at baseline and 12 months post enrollment.

All adverse events related to the interventions will be recorded and monitored until resolved. Medical personnel will decide if continued trial participation is feasible based on these reports, with the final decision made by the participant.

Data Collection

A clinical research co-ordinator (CRC) will collect information on demographic characteristics such as: gender, age, vital signs, height and weight and medication history. The patients' medical history will be taken along with pacemaker data (implant date, implanted device, programmed pacemaker mode, base rate, and cumulative pacing percentages), and blood draw will be obtained. Biological specimens will be analysed and stored in the on-site research pathology laboratory. Patients randomised to intervention will undergo a baseline transthoracic echocardiogram which will be assessed by the CRC. All data will be stored anonymously and will be locked once complete.

For peer review only

For peer review only - http://bmjopen.bmj.com/site/about/guidelines.xhtml

To ensure protocol adherence, a single research team will undertake all study-specific activity and be regularly monitored.

Digital follow-up will occur at 12 month post recruitment to record mortality and hospitalisation data and changes to medical therapy. Patient data regarding cardiac transplatation or device upgrade will be recorded and subsequently reported.

Sample size

The trial is designed to detect a reduction in hospitalisation or mortality rate of 7.5% at one year in patients identified with cardiac dysfunction from 15% anticipated in patients randomised to the standard pathway [9]. Given approximately one third of patients in both randomised arms are estimated to have cardiac dysfunction, a 7.5% reduction would be diluted to a 9% overall reduction in the enhanced pathway arm. To detect a reduction in events from 15% to 9% (equivalent to a hazard ratio equal to 0.58) using log-rank analysis with an overall type 1 error rate of 0.05 (two-sided analysis) and a power of 0.90, requires a total of 146 events to be observed in at least 1070 participants (nQuery Advisor assuming 18 month recruitment) inflated to 1200 in anticipation of minimal drop-out.

Statistical Analysis Plan

Primary analysis will be performed using the pre-specified endpoints in the intention-to-treat population (all randomised patients). . Time to event will be summarised across randomised groups using Kaplan-Meier estimates, hazard ratios and comparison across groups using log-rank tests, reporting 12 month survival.

For peer review only

For peer review only - http://bmjopen.bmj.com/site/about/guidelines.xhtml

Secondary analyses will be based on estimating adjusted treatment effects through Cox Proportional Hazards regression model the influence of baseline patient characteristics on outcome. These variables include age, gender, site, co-morbidities, type of device, NYHA class, medication, blood measures and underlying rhythm. A post-hoc exploratory cost-effectiveness analysis will be conducted.

Quality of life data is collected at 2 time points and will be summarised graphically, conditional on patient survival. Quality adjusted time to event analysis may be appropriate to report quality adjusted estimates of 12-month survival.

External validation of the risk model will be based on fitting the published model to the OPT-PACE trial data to report its goodness of fit. Data will be pooled, and stratified by study, to remodel parameter estimates with the aim of improving precision.

Patient and Public Involvement

The research question developed as a large number of pacemaker patients were seen in HF clinic with deterioration in their ventricular function and symptoms who were followed-up annually in pacemaker clinic and yet, these had not been detected. The study was initially discussed with a well-established local patient and public involvement (PPI) advisory group (AG) consisting of cardiovascular patients and their families. The AG particularly felt that undergoing all study assessments in one day was appealing to potential participants but that the intervention was not a large burden and that it was more important to know the effect on patient survival and the number of times patients were hospitalised, not just whether there was an improvement in heart function from optimised care in terms of outcome

For peer review only - http://bmjopen.bmj.com/site/about/guidelines.xhtml

measures. Once the final protocol was established, it was reviewed by the PPI-AG along with patient information sheets to ensure the plain English was at an appropriate level. Regular updates will be presented regarding recruitment and any adverse events. Study results will be discussed and an appropriate method of dissemination will be designed together between the AG and researchers to deliver these to participants.

Ethics and Dissemination

The trial design and protocol have received ethical approval from the South Yorkshire research ethics committee (12/YH/0487) and is registered on Clinicaltrials.gov (NCT01819662). Amendments will be documented.

Trial results will be communicated on a local level to healthcare professionals and patient involvement groups. Results will be published in international peer-reviewed journals with authorship as determined by the international committee of medical journal editors' guidelines [30] and highlighted using social media campaigns. The National Institute of Health Research will be made aware prior to publication but no publication restrictions apply.

Discussion

Patients with permanent pacemakers are at increased risk of prevalent heart failure with a reduced quality of life and incident HF leading to hospitalisations and a poorer prognosis. Despite increasing recognition of this, and the development of device-based options to limit unnecessary ventricular pacing, patients with permanent pacemakers rarely undergo any assessment to identify prevalent HF or their future risk of HF. Pacemaker follow-up services have remained resolutely technical with intervals based upon historical risk of pacemaker

For peer review only

For peer review only - http://bmjopen.bmj.com/site/about/guidelines.xhtml

device failure rather than focusing on patient requirements in the face of negligible pacemaker failure rates and extended battery longevity.

A validated tool combining clinical and technical data by which patients' risk of the one major complication of pacemaker therapy - heart failure - could be assessed, allowing individualised follow-up intervals has the potential to substantially improve patient experience whilst also offering the opportunity to focus screening and lead to the initiation of optimal therapy. Although there are several clinical and pacemaker-related features linked to the presence of cardiac dysfunction, no validated risk score exists to select those that should be referred for assessment.

The management pathways used in this trial will offer individualised healthcare, and establish the relative benefits of primary, secondary and tertiary expertise and interventions. Our previous data have shown that reprogramming to avoid RV pacing is safe and well tolerated by patients. Combining this with medical therapy for HF in collaboration with a HF service as a new model of care for people with pacemakers is feasible and could improve their quality of life and overall prognosis whilst also being highly cost-effective. We aim to test the clinical effectiveness of this approach when applied to a large number of patients at multiple sites.

Conclusions

OPT-PACE is an important randomised controlled trial, supported by non-industry funding, that will investigate whether assessing left ventricular function to inform pacemaker programming and medical decisions, can improve the morbidity and mortality in pacemaker patients, compared with usual care.

For peer review only

For peer review only - http://bmjopen.bmj.com/site/about/guidelines.xhtml

The results will have implications for the stratification of pacemaker patients and the organisation of diagnostic and therapeutic pathways to reduce the incidence of HF in this population.

Abbreviations

BR: Base rate; CHF: Chronic heart failure; EQ-5D: EuroQol 5-Dimension; HF: Heart failure; LV: Left ventricle; LVEF: Left ventricular ejection fraction; LVSD: Left ventricular systolic dysfunction; NT-proBNP: N-terminal prohormone of brain natriuretic peptide; RV: Right ventricular

Authors Contributions

KW MFP JG MTK and RMC researched the topic and devised the study. MP and KW provided the first draft of the manuscript. DDS and DAC provide statistical oversight and RGG managed data storage and aided analysis. MFP JG HAJ JEL RB HC RMC MTK and KW contributed equally to data collection and all authors contributed to manuscript preparation.

Acknowledgements

The authors would like to express their appreciation for the valuable contributions of participants with a pacemaker who are, or will, participate in this trial, the PPI-AG for their practical and insightful suggestions, and for the consistent administrative support of Mrs Andrea Marchant and Miss Lisa Trueman.

Funding Statement

For peer review only

For peer review only - http://bmjopen.bmj.com/site/about/guidelines.xhtml

The research is supported by the National Institute for Health Research (NIHR) infrastructure at Leeds, and a NIHR clinician scientist fellowship (NIHR-CS-2012-032). The views expressed are those of the author(s) and not necessarily those of the NHS, the NIHR or the Department of Health. This research was supported by the National Institute for Health Research Leeds Clinical Research Facility.

Endpoint Review Committee

Members: KW, MTK, MP, JF, 2 heart failure specialist nurses

Competing Interests Statement

MFP holds a NIHR clinical doctoral fellowship outside of this work. JG holds a NIHR postdoctoral fellowship outside of this work. RMC holds a fellowship with the BHF. MTK holds a BHF chair. KKW holds an NIHR clinician scientist award.

Figure Legend

Figure 1: Ventricular pacing avoidance protocol [9]

Figure 2: OPT-PACE study protocol

References

For peer review only

1. Epstein, A.E., et al., *2012 ACCF/AHA/HRS focused update incorporated into the ACCF/AHA/HRS 2008 guidelines for device-based therapy of cardiac rhythm abnormalities.* Circulation, 2013. **127**(3): p. e283-e352.
2. Shaw, D.B., et al., *Survival in second degree atrioventricular block.* Heart, 1985. **53**(6): p. 587-593.
3. Lamas, G.A., et al., *Quality of life and clinical outcomes in elderly patients treated with ventricular pacing as compared with dual-chamber pacing.* New England Journal of Medicine, 1998. **338**(16): p. 1097-1104.
4. Lee, M.A., et al., *Effects of long-term right ventricular apical pacing on left ventricular perfusion, innervation, function and histology.* Journal of the American College of Cardiology, 1994. **24**(1): p. 225-232.
5. Begg, G., et al., *154 Patients receiving standard pacemaker generator replacements frequently have impaired left ventricular function and exercise intolerance, related to the percentage of right ventricular pacing.* Heart, 2011. **97**(Suppl 1): p. A86-A86.
6. Sweeney, M.O., et al., *Adverse effect of ventricular pacing on heart failure and atrial fibrillation among patients with normal baseline QRS duration in a clinical trial of pacemaker therapy for sinus node dysfunction.* Circulation, 2003. **107**(23): p. 2932-2937.
7. Thackray, S.D., et al., *The prevalence of heart failure and asymptomatic left ventricular systolic dysfunction in a typical regional pacemaker population.* European Heart Journal, 2003. **24**(12): p. 1143-1152.
8. ZHANG, X.H., et al., *New-onset heart failure after permanent right ventricular apical pacing in patients with acquired high-grade atrioventricular block and normal left ventricular function.* Journal of cardiovascular electrophysiology, 2008. **19**(2): p. 136-141.
9. Gierula, J., et al., *Pacing-associated left ventricular dysfunction? Think reprogramming first!* Heart, 2014. **100**(10): p. 765-769.
10. Nieminen, M.S., et al., *EuroHeart Failure Survey II (EHFS II): a survey on hospitalized acute heart failure patients: description of population.* European heart journal, 2006. **27**(22): p. 2725-2736.
11. Gierula, J., et al., *Patients with long-term permanent pacemakers have a high prevalence of left ventricular dysfunction.* Journal of Cardiovascular Medicine, 2015. **16**(11): p. 743-750.
12. Shen, W.-K., et al., *Long-term survival after pacemaker implantation for heart block in patients≥ 65 years.* The American journal of cardiology, 1994. **74**(6): p. 560-564.
13. JELIĆ, V., et al., *Survival in 1,431 pacemaker patients: prognostic factors and comparison with the general population.* Pacing and Clinical Electrophysiology, 1992. **15**(2): p. 141-147.
14. Mayosi, B.M., F. Little, and R.N.S. MILLAR, *Long-term survival after permanent pacemaker implantation in young adults: 30 year experience.* Pacing and clinical electrophysiology, 1999. **22**(3): p. 407-412.
15. Sweeney, M.O., et al., *Reduced ejection fraction, sudden cardiac death, and heart failure death in the mode selection trial (MOST): implications for device selection in elderly patients with sinus node disease.* Journal of cardiovascular electrophysiology, 2008. **19**(11): p. 1160-1166.

For peer review only

For peer review only - http://bmjopen.bmj.com/site/about/guidelines.xhtml

16. Shen, W.-K., et al., *Survival and functional independence after implantation of a permanent pacemaker in octogenarians and nonagenarians: a population-based study.* Annals of internal medicine, 1996. **125**(6): p. 476-480.
17. Brunner, M., et al., *Long-term survival after pacemaker implantation: Prognostic importance of gender and baseline patient characteristics.* European Heart Journal, 2004. **25**(1): p. 88-95.
18. Jahangir, A., et al., *Relation between mode of pacing and long-term survival in the very elderly.* Journal of the American College of Cardiology, 1999. **33**(5): p. 1208-1216.
19. Braunschweig, F., M.R. Cowie, and A. Auricchio, *What are the costs of heart failure?* Europace, 2011. **13**(suppl_2): p. ii13-ii17.
20. Purdy, S., et al., *Ambulatory care sensitive conditions: terminology and disease coding need to be more specific to aid policy makers and clinicians.* Public health, 2009. **123**(2): p. 169-173.
21. Kolb, C., et al., *Reduction of right ventricular pacing with advanced atrioventricular search hysteresis: results of the PREVENT study.* Pacing and Clinical Electrophysiology, 2011. **34**(8): p. 975-983.
22. Sweeney, M., *Search AV Extension and Managed Ventricular Pacing for Promoting Atrioventricular Conduction (SAVE PACe) Trial. Minimizing ventricular pacing to reduce atrial fibrillation in sinus-node disease.* N Engl J Med, 2007. **357**: p. 1000-1008.
23. DAVY, J., et al., *Near elimination of ventricular pacing in SafeR mode compared to DDD modes: a randomized study of 422 patients.* Pacing and clinical electrophysiology, 2012. **35**(4): p. 392-402.
24. Marinskis, G., et al., *Practices of cardiac implantable electronic device follow-up: results of the European Heart Rhythm Association survey.* Europace, 2012. **14**(3): p. 423-425.
25. Lang, R.M., et al., *Recommendations for cardiac chamber quantification by echocardiography in adults: an update from the American Society of Echocardiography and the European Association of Cardiovascular Imaging.* Journal of the American Society of Echocardiography, 2015. **28**(1): p. 1-39. e14.
26. Excellence, N.I.f.H.a.C., *Dual-chamber pacemakers for symptomatic bradycardia due to sick sinus syndrome without atrioventricular block (TA324)*. 2014, NICE: London.
27. NICE, *Dual-chamber pacemakers for symptomatic bradycardia due to sick sinus syndrome and/or atrioventricular block*. 2017.
28. Excellence, N.I.f.H.a.C., *Chronic heart failure in adults: management*. 2010, NICE: London.
29. Higgins, J.P.T., S. Green, and Cochrane Collaboration., *Cochrane handbook for systematic reviews of interventions*. 2011, Cochrane Collaboration: [Oxford]. p. 1 online resource.
30. Editors, I.C.o.M.J. *Defining the role of authors and contributors*. 2018 [cited 2018 01/02/2018]; Available from: http://www.icmje.org/recommendations/browse/roles-and-responsibilities/defining-the-role-of-authors-and-contributors.html.

For peer review only

For peer review only - http://bmjopen.bmj.com/site/about/guidelines.xhtml

Figure 1. Right ventricular avoidance algorithm

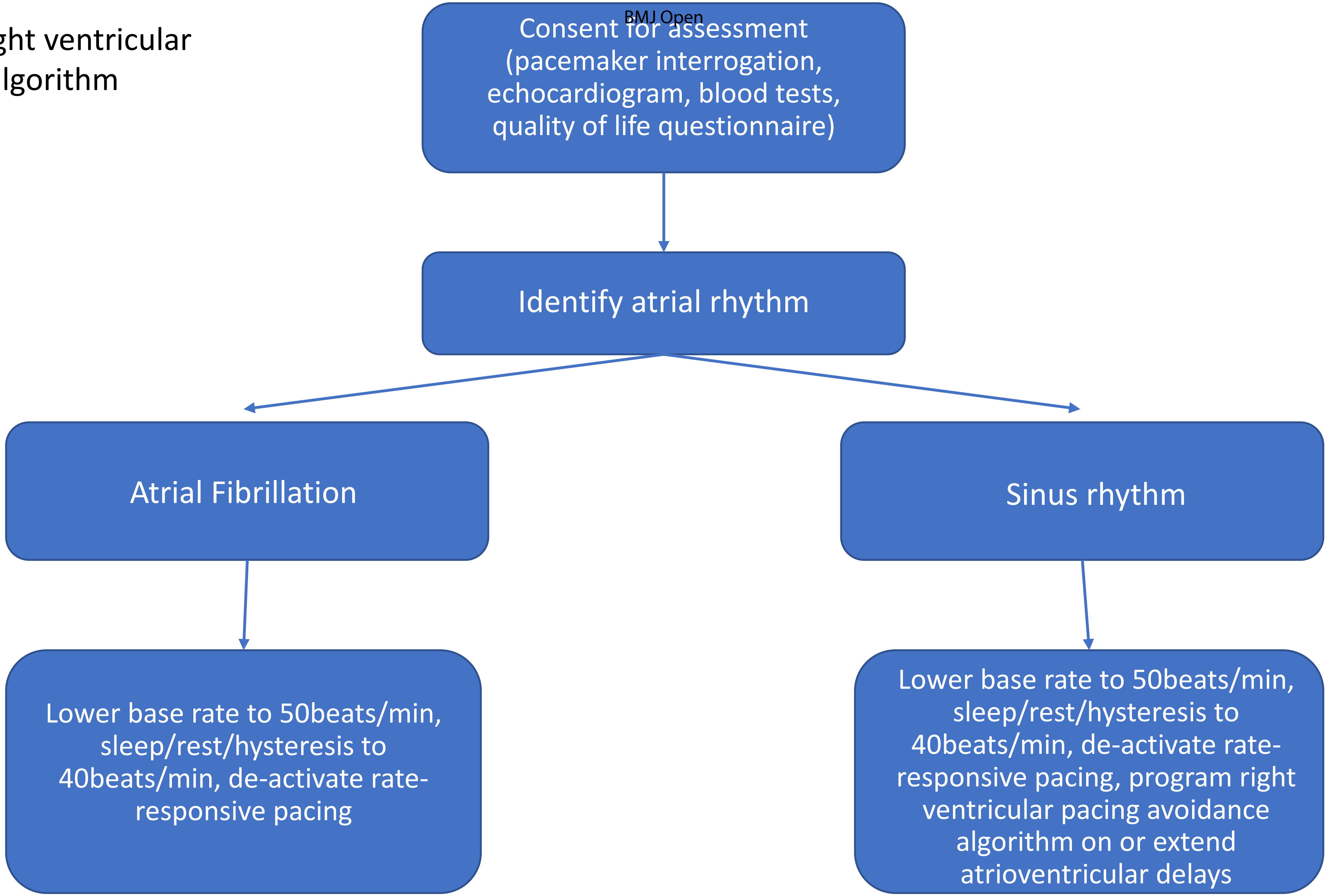

Figure 2. OPT-PACE study protocol

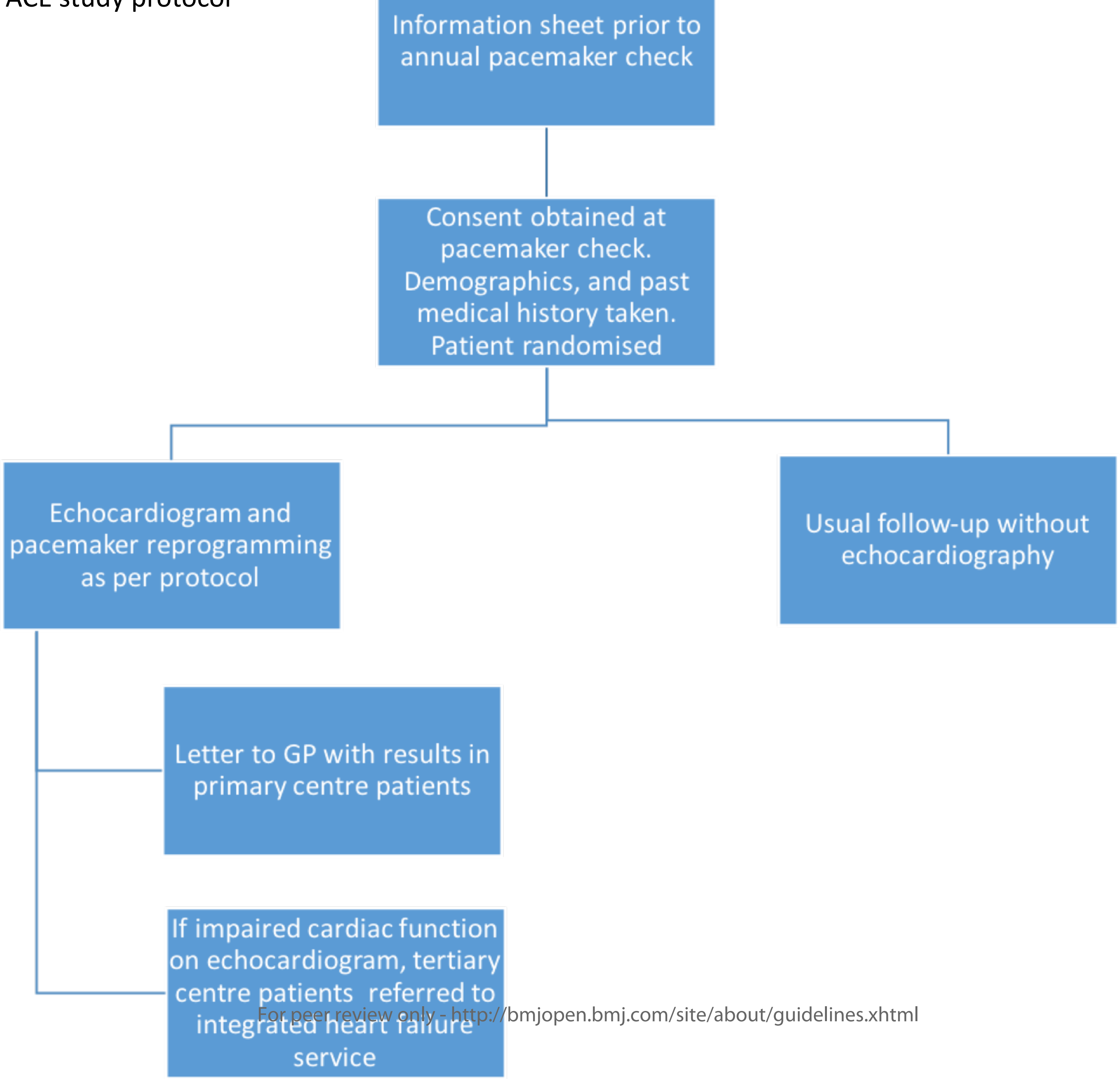

For peer review only - http://bmjopen.bmj.com/site/about/guidelines.xhtml

STANDARD PROTOCOL ITEMS: RECOMMENDATIONS FOR INTERVENTIONAL TRIALS

SPIRIT 2013 Checklist: Recommended items to address in a clinical trial protocol and related documents*

Section/item	Item No	Description	Addressed on page number
**Administrative information**			
Title	1	Descriptive title identifying the study design, population, interventions, and, if applicable, trial acronym	1
Trial registration	2a	Trial identifier and registry name. If not yet registered, name of intended registry	1
	2b	All items from the World Health Organization Trial Registration Data Set	1,
Protocol version	3	Date and version identifier	8
Funding	4	Sources and types of financial, material, and other support	1, 8, 15
Roles and responsibilities	5a	Names, affiliations, and roles of protocol contributors	1, 15
	5b	Name and contact information for the trial sponsor	1
	5c	Role of study sponsor and funders, if any, in study design; collection, management, analysis, and interpretation of data; writing of the report; and the decision to submit the report for publication, including whether they will have ultimate authority over any of these activities	N/A
	5d	Composition, roles, and responsibilities of the coordinating centre, steering committee, endpoint adjudication committee, data management team, and other individuals or groups overseeing the trial, if applicable (see Item 21a for data monitoring committee)	11, 15

For peer review only

For peer review only - http://bmjopen.bmj.com/site/about/guidelines.xhtml

Introduction

Background and rationale	6a	Description of research question and justification for undertaking the trial, including summary of relevant studies (published and unpublished) examining benefits and harms for each intervention	4-8
	6b	Explanation for choice of comparators	4-7, 10
Objectives	7	Specific objectives or hypotheses	7
Trial design	8	Description of trial design including type of trial (eg, parallel group, crossover, factorial, single group), allocation ratio, and framework (eg, superiority, equivalence, noninferiority, exploratory)	4-9

Methods: Participants, interventions, and outcomes

Study setting	9	Description of study settings (eg, community clinic, academic hospital) and list of countries where data will be collected. Reference to where list of study sites can be obtained	8,9
Eligibility criteria	10	Inclusion and exclusion criteria for participants. If applicable, eligibility criteria for study centres and individuals who will perform the interventions (eg, surgeons, psychotherapists)	8,9
Interventions	11a	Interventions for each group with sufficient detail to allow replication, including how and when they will be administered	9, 11
	11b	Criteria for discontinuing or modifying allocated interventions for a given trial participant (eg, drug dose change in response to harms, participant request, or improving/worsening disease)	8, 9, 11
	11c	Strategies to improve adherence to intervention protocols, and any procedures for monitoring adherence (eg, drug tablet return, laboratory tests)	11
	11d	Relevant concomitant care and interventions that are permitted or prohibited during the trial	NA
Outcomes	12	Primary, secondary, and other outcomes, including the specific measurement variable (eg, systolic blood pressure), analysis metric (eg, change from baseline, final value, time to event), method of aggregation (eg, median, proportion), and time point for each outcome. Explanation of the clinical relevance of chosen efficacy and harm outcomes is strongly recommended	10,11
Participant timeline	13	Time schedule of enrolment, interventions (including any run-ins and washouts), assessments, and visits for participants. A schematic diagram is highly recommended (see Figure)	8,9, Figure 2

For peer review only - http://bmjopen.bmj.com/site/about/guidelines.xhtml

Sample size	14	Estimated number of participants needed to achieve study objectives and how it was determined, including clinical and statistical assumptions supporting any sample size calculations	11
Recruitment	15	Strategies for achieving adequate participant enrolment to reach target sample size	11-12

Methods: Assignment of interventions (for controlled trials)

Allocation:			
Sequence generation	16a	Method of generating the allocation sequence (eg, computer-generated random numbers), and list of any factors for stratification. To reduce predictability of a random sequence, details of any planned restricton (eg, blocking) should be provided in a separate document that is unavailable to those who enrol participants or assign interventions	8,9
Allocation concealment mechanism	16b	Mechanism of implementing the allocation sequence (eg, central telephone; sequentially numbered, opaque, sealed envelopes), describing any steps to conceal the sequence until interventions are assigned	8,9
Implementation	16c	Who will generate the allocation sequence, who will enrol participants, and who will assign participants to interventions	5-8
Blinding (masking)	17a	Who will be blinded after assignment to interventions (eg, trial participants, care providers, outcome assessors, data analysts), and how	9
	17b	If blinded, circumstances under which unblinding is permissible, and procedure for revealing a participant's allocated intervention during the trial	9,13

Methods: Data collection, management, and analysis

Data collection methods	18a	Plans for assessment and collection of outcome, baseline, and other trial data, including any related processes to promote data quality (eg, duplicate measurements, training of assessors) and a description of study instruments (eg, questionnaires, laboratory tests) along with their reliability and validity, if known. Reference to where data collection forms can be found, if not in the protocol	9
	18b	Plans to promote participant retention and complete follow-up, including list of any outcome data to be collected for participants who discontinue or deviate from intervention protocols	11

Data management	19	Plans for data entry, coding, security, and storage, including any related processes to promote data quality (eg, double data entry; range checks for data values). Reference to where details of data management procedures can be found, if not in the protocol	8-11
Statistical methods	20a	Statistical methods for analysing primary and secondary outcomes. Reference to where other details of the statistical analysis plan can be found, if not in the protocol	12
	20b	Methods for any additional analyses (eg, subgroup and adjusted analyses)	12
	20c	Definition of analysis population relating to protocol non-adherence (eg, as randomised analysis), and any statistical methods to handle missing data (eg, multiple imputation)	12
**Methods: Monitoring**			
Data monitoring	21a	Composition of data monitoring committee (DMC); summary of its role and reporting structure; statement of whether it is independent from the sponsor and competing interests; and reference to where further details about its charter can be found, if not in the protocol. Alternatively, an explanation of why a DMC is not needed	13
	21b	Description of any interim analyses and stopping guidelines, including who will have access to these interim results and make the final decision to terminate the trial	12
Harms	22	Plans for collecting, assessing, reporting, and managing solicited and spontaneously reported adverse events and other unintended effects of trial interventions or trial conduct	11
Auditing	23	Frequency and procedures for auditing trial conduct, if any, and whether the process will be independent from investigators and the sponsor	11
**Ethics and dissemination**			
Research ethics approval	24	Plans for seeking research ethics committee/institutional review board (REC/IRB) approval	11, 12
Protocol amendments	25	Plans for communicating important protocol modifications (eg, changes to eligibility criteria, outcomes, analyses) to relevant parties (eg, investigators, REC/IRBs, trial participants, trial registries, journals, regulators)	13

Consent or assent	26a	Who will obtain informed consent or assent from potential trial participants or authorised surrogates, and how (see Item 32)	9,11
	26b	Additional consent provisions for collection and use of participant data and biological specimens in ancillary studies, if applicable	N/A
Confidentiality	27	How personal information about potential and enrolled participants will be collected, shared, and maintained in order to protect confidentiality before, during, and after the trial	11
Declaration of interests	28	Financial and other competing interests for principal investigators for the overall trial and each study site	15,16
Access to data	29	Statement of who will have access to the final trial dataset, and disclosure of contractual agreements that limit such access for investigators	11
Ancillary and post-trial care	30	Provisions, if any, for ancillary and post-trial care, and for compensation to those who suffer harm from trial participation	8
Dissemination policy	31a	Plans for investigators and sponsor to communicate trial results to participants, healthcare professionals, the public, and other relevant groups (eg, via publication, reporting in results databases, or other data sharing arrangements), including any publication restrictions	12,13
	31b	Authorship eligibility guidelines and any intended use of professional writers	15
	31c	Plans, if any, for granting public access to the full protocol, participant-level dataset, and statistical code	No plans
**Appendices**			
Informed consent materials	32	Model consent form and other related documentation given to participants and authorised surrogates	N/A can be provided on request
Biological specimens	33	Plans for collection, laboratory evaluation, and storage of biological specimens for genetic or molecular analysis in the current trial and for future use in ancillary studies, if applicable	11,12,13

*It is strongly recommended that this checklist be read in conjunction with the SPIRIT 2013 Explanation & Elaboration for important clarification on the items. Amendments to the protocol should be tracked and dated. The SPIRIT checklist is copyrighted by the SPIRIT Group under the Creative Commons "Attribution-NonCommercial-NoDerivs 3.0 Unported" license.

For peer review only - http://bmjopen.bmj.com/site/about/guidelines.xhtml

For peer review only

For peer review only - http://bmjopen.bmj.com/site/about/guidelines.xhtml
